# Repression of let-7a cluster prevents adhesion of colorectal cancer cells by enforcing a mesenchymal phenotype in presence of liver inflammation

**DOI:** 10.1038/s41419-018-0477-1

**Published:** 2018-04-25

**Authors:** Lipeng Cheng, Li Geng, Binghua Dai, Tao Zheng, Jun Fu, Liang Qiao, Wenchang Cai, Yue Wang, Jiamei Yang

**Affiliations:** 1 Department of Special Treatment and Liver Transplantation, Shanghai Eastern Hepatobiliary Surgery Hospital, Second Military Medical University, Shanghai, 200438 China; 2 Department of General Surgery, People’s Liberation Army Nanjing General Hospital, Nanjing, 210002 China; 3 Department of Hepatobiliary Pancreatic and Spleen Surgery, Xuzhou Central Hospital, Xuzhou, 221000 China; 4 Research Center of Developmental Biology, Second Military Medical University, Shanghai, 200433 China

## Abstract

The liver is the most common site of metastasis in patients with colorectal cancer, and colorectal cancer liver metastasis (CRLM) is associated with poor rates of survival. However, CRLM occurs infrequently in livers exhibiting signs of hepatitis or cirrhosis, suggesting a role for inflammation in attenuating CRLM. The molecular mechanisms driving this phenomenon remain unclear. The aim of this study was to confirm the mechanism by which liver inflammation inhibits CRLM. We used BALB/c animal models of inflammatory liver diseases to confirm that liver inflammation inhibits CRLM, and then elucidated the molecular mechanisms governing that process. Out data showed that liver inflammation induces IFN-γ expression, which then downregulates expression of the let-7a cluster through IRF-1 in colorectal cancer cells. Finally, we showed that modulation of let-7a expression regulated the epithelial–mesenchymal transition in colorectal cancer cell lines, and inhibited their capacity to metastasize in vivo. Cumulatively, we clarified the critical role played by the IFN-γ/IRF-1/let-7a cluster/EMT pathway in regulating the spread of circulating colorectal cancer cells to the liver, and highlighted the critical role that the hepatitis microenvironment plays in modulating that process.

## Introduction

Colorectal cancer (CRC) has become the third most common type of cancer worldwide and the fourth most common cause of cancer-related mortality^1,2^. CRC is closely linked to a Western lifestyle, gender, and increased age^3^.

Tumor recurrence and distant metastasis are still the main causes of death in CRC patients^4^. Clinically, the liver is the most common site of distant metastasis in patients with CRC. About 18.8% of CRC patients presented with synchronous liver metastases at diagnosis and 29.3% developed metachronous liver metastases within 3 years^5,6^.

Interestingly, CRC patients with pathological liver disease were noted to have a lower incidence of colorectal cancer liver metastasis (CRLM)^7–18^. Numerous theories have been proposed in an effort to explain this clinical feature, including activation of liver-associated immunity^10^, fibrosis, and sinusoid capillarization which may induce an unfavorable hepatic “soil” in cirrhotic liver microenvironment^19,20^, and the recruitment of adaptive immune cells which may constrain CRLM through perforin-dependent or Fas/FasL mechanisms^21–25^ and through the production of cytokines, such as interferon-γ (IFN-γ) and tumor necrosis factor-α (TNF-α)^26,27^. However, there is still no consensus as to what molecular mechanisms underlie the inverse relationship between pathological liver diseases and the presentation of CRLM.

MicroRNAs (miRNA), one of the endogenous non-coding RNAs, regulate the expression of about 60% of human genes, especially oncogenes, by enhancing mRNA degradation. Multiple lines of research has demonstrated that miRNAs are involved in regulating the development of CRLM by suppressing the generation of metastasis-related metabolites^28,29^, promoting stemness and metastatic potential^30^ and regulating the epithelial–mesenchymal transition (EMT)^31–33^ of CRC cells.

MiRNA let-7, comprised of 12 members, was first identified as a regulator of *Caenorhabditis elegans* development^34^ and has been reported as a tumor suppressor in many kinds of malignancies including CRC^35,36^. Our previous work has demonstrated that the biogenesis of let-7a-1-5p, let-7d-5p, let-7f-1-5p (let-7a cluster) was strongly inhibited by stimulation of IFN-γ in colorectal cancer cells HT29 and sensitized the cells to Fas-related apoptosis^37^.

In this study, we aimed to confirm that IRF-1-mediated downregulation of the let-7a cluster in the inflammatory microenvironment might prevent the circulating CRC cells from adhering to and colonizing the liver by enforcing the mesenchymal characteristics of the CRC cells.

## Results

More than 10 retrospective studies (Table 1) have reported that CRLM occurs infrequently in CRC patients with pathological liver diseases, such as chronic hepatitis infection, cirrhosis, fatty liver, and the other chronic liver diseases^7–17^.

**Table 1:** Previous series of liver metastases in pathological livers

	Country	Cases (*n*)	LM in NL (%)	LM in PL (%)	*P-*value	Liver disease
Hamaya et al.^7^	Japan	240,377	43.20	26.30	>0.05	Cirrohosis
Vanbockrijck^8^	Belgium	2162	46.40	33.30	<0.01	Cirrohosis
Hayashi et al.^9^	Japan	839	16.02	1.65	<0.01	Fatty liver
Ustunomiya et al. (1999)^13^	Japan	438	21.20	8.10	>0.05	Infection HBV/HCV
Song et al.^10^	China	512	27.10	13.50	<0.05	Infection HBV
Lascone et al. (2005)^15^	Belgium	747	32.00	4.70	<0.05	Cirrhosis
Qian et al.^12^	China	2352	23.47	7.86	<0.01	Hepatitis+cirrosis
Qiu et al.^11^	China	1298	28.16	14.16	<0.01	Hepatitis infection
Wang et al.^17^	China	354	16.90	2.6	<0.01	Hepatitis infection
Zeng et al.^18^	China	2868	18.64	8.85	<0.01	Hepatitis infection
Murono et al.^16^	Japan	604	9.61	3.17	>0.05	Fatty liver

*LM* liver metastasis, *NL* normal liver, *PL* pathological liver, *HBV* hepatitis B virus, *HCV* hepatitis C virus

### The inflammatory liver microenvironment prevents colorectal liver metastasis

**Fig. 1:**
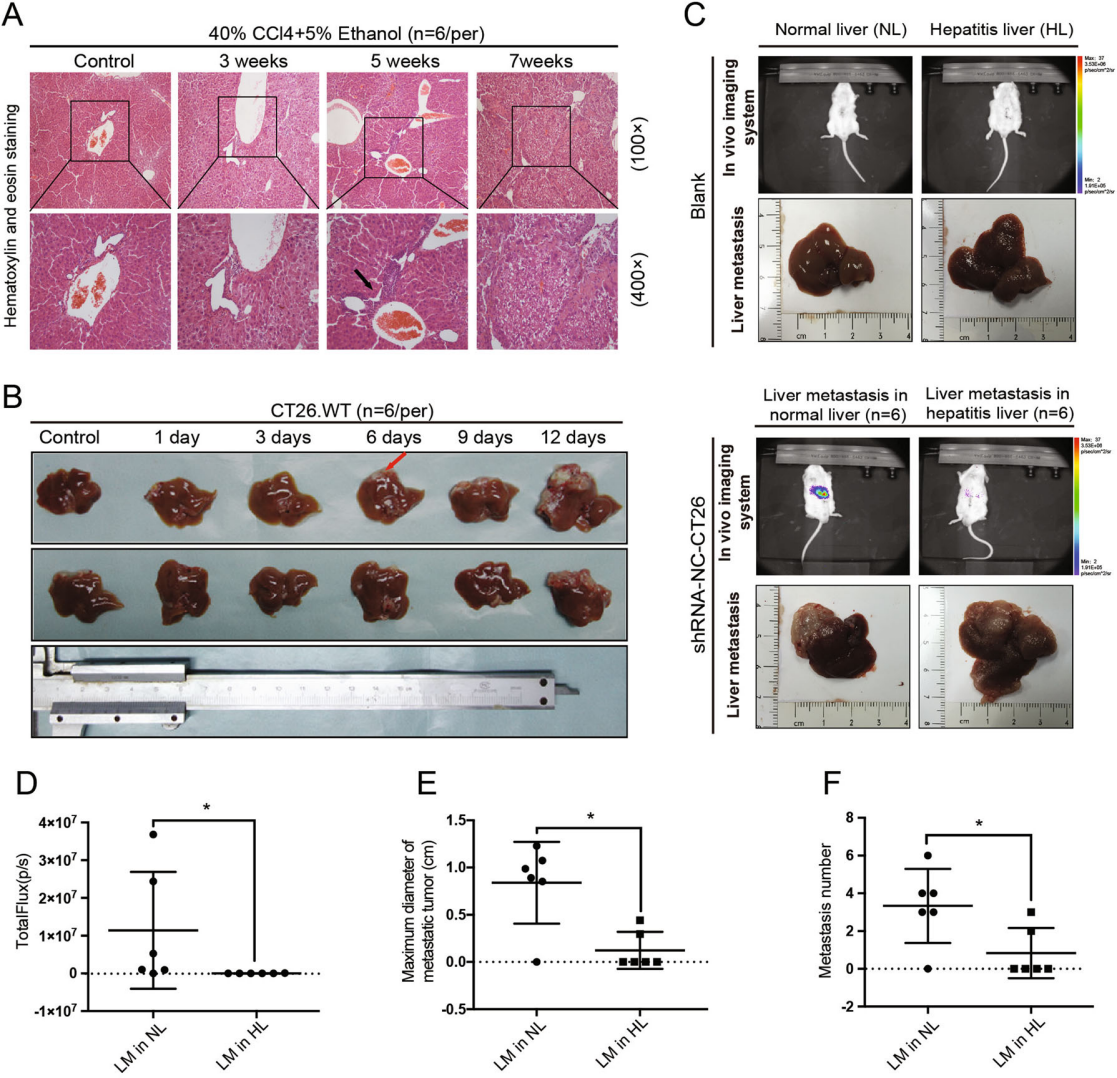
Liver inflammatory environment reduced the development of colorectal cancer liver metastasis. **a** Six animal models of liver inflammation were constructed by administering a 40% carbon tetrachloride (CCl_4_) gavage and having the animals drink 5% alcohol. Histopathological characteristics of inflamed liver specimens were observed after hematoxylin and eosin (H&E) staining. Lymphocytic infiltration became more common in the portal areas (indicated by the black arrow) after 5 weeks of model induction. **b** Colorectal liver metastasis models (*n* = 6) were constructed by splenic injection of CT26. WT cells within different time periods; after which the final modeling time was determined. Hepatic metastatic foci (indicated by the red arrow) were observed as early as 6 days, and by day 12, >80%(5/6) of mice exhibited signs of liver metastases. **c**, **d** Whole-body fluorescence imaging and hepatectomy were performed to detect the incidence of CRLM. Excised liver specimens showed that hepatitis led to decreases in liver tissue smoothness and glossiness. In addition, less powerful fluorescence was detected in the right upper quadrants of mice with hepatitis (*P* < 0.05), and liver metastases of CT26.WT cells were rarely present in that group (2/6 vs. 5/6, *P* = 0.079). **e**, **f** The number of metastases (*P* < 0.05) and maximum diameters of the metastatic tumors (*P* < 0.05) were dramatically lower in the mice with hepatitis. NL normal liver, HL hepatitis liver, LM liver metastasis. The Chi-square test was used to test the rates of incidence of metastasis. Nonparametric test was used to test the total powerful of fluorescence, the number of metastatic foci, and the maximum diameter of metastasis. Values are shown as the mean ± SD. *P < 0.05; **P < 0.001

Animal models of liver inflammation were constructed by gavaging animals for 5 weeks with 40% carbon tetrachloride (CCl_4_). Hematoxylin and eosin (H&E) staining revealed the infiltration of inflammatory cells into the livers of those mice. The infiltrated cells (predominantly lymphocytes) were mostly distributed in the portal areas, and particularly around the interlobular artery and interlobular bile duct. After 7 weeks of gavaging, hepatic lobe reconstruction and typical pseudo-lobule formation were identified (Fig. 1a).

CRLM animal models were constructed by injecting CT26 cells into the spleens of BALB/c mice (*n* = 6). A whole-body in vivo imaging system was used to detect the incidence of CRLM. The results showed that hepatic metastatic foci could be observed as early as 6 days after model construction (metastatic rate was 16.7%), and by day 12, 83.3% of the mice exhibited signs of liver metastases (Fig. 1b).

To test whether the pathologic liver diseases induced in our inflammatory model could abrogate CRLM, shRNA-NC-CT26 cells were injected into BALB/c mice (*n* = 6) which had or had not been subjected to liver inflammation. Twelve days later, all of the mice were intraperitoneally injected with luciferin, and whole-body in vivo was performed to detect the incidence of CRLM. Specimens of liver tissue were studied both with the naked eye and under a microscope, and results revealed liver metastases of colorectal cancer (Fig. 1c). In a group of normal mice, 83.3% (5/6) had hepatic metastases, compared to only 33.3%(2/6) in the inflammation group (*P* = 0.079). We detected less powerful total fluorescence in the mice with hepatitis (*P* < 0.05). In addition, the maximum diameters of metastatic tumors (*P* < 0.05) and the numbers of metastases (*P* < 0.05) were dramatically lower in the mice with hepatitis when compared with mice in the normal control group.

### IFN-γ may play an important role in determining the lower incidence of CRLM in an inflammatory environment

**Fig. 2:**
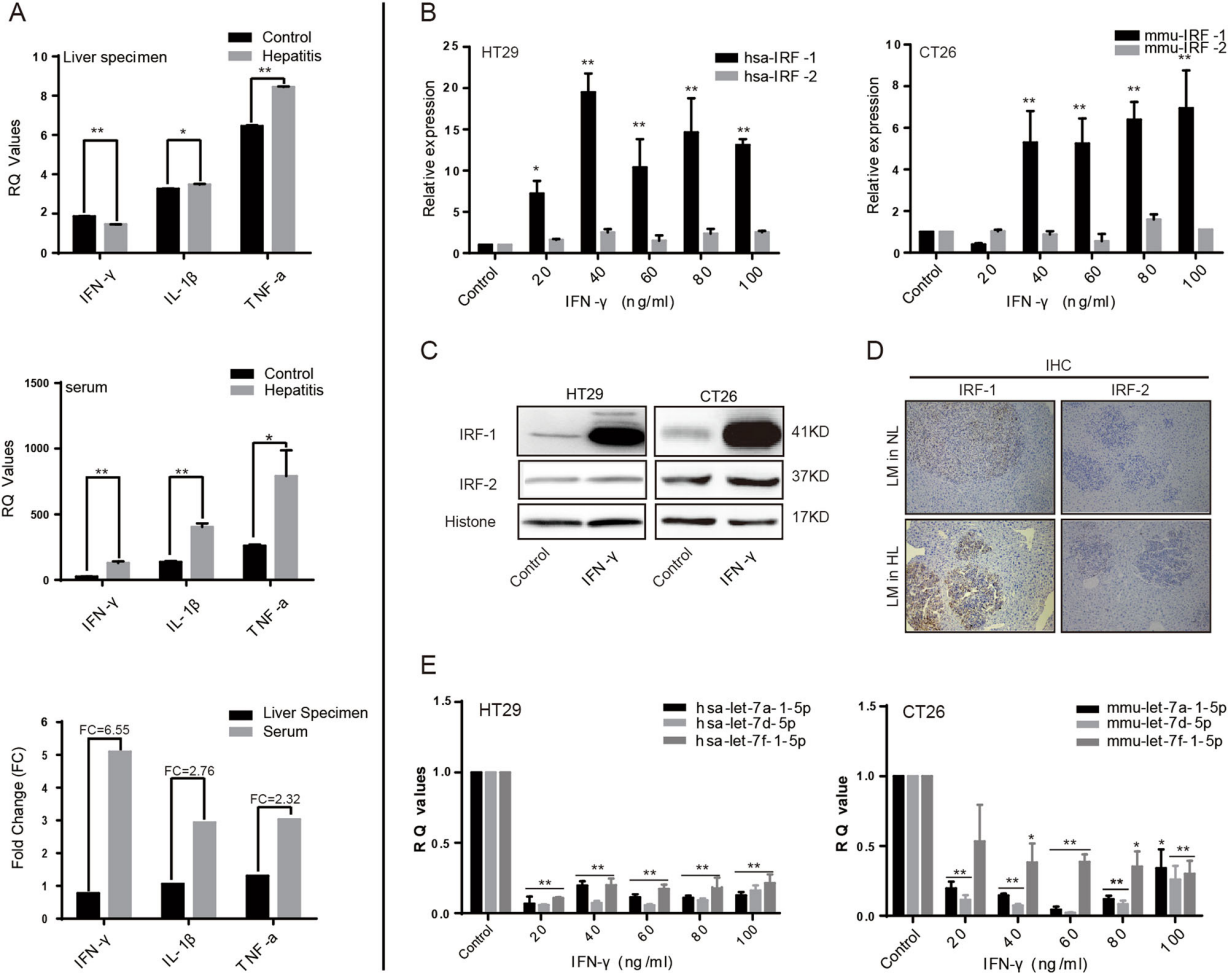
There was an inverse correlation between expression of let-7a cluster and IRF-1 in the inflamed livers. **a** ELISA assay was used to detect the expression of such pro-inflammatory factors as IFN-γ, TNF-α, and IL-1β in liver specimens and corresponding serum sampled from the mice (*n* = 6) with hepatitis. We found that some slight differences in the expression of those three factors in liver specimens without reaching statistical significance. However, the expression of IFN-γ (*P* < 0.01), TNF-α (*P* < 0.05), and IL-1β (*P* < 0.01) were significantly increased in the serum of mice with hepatitis, particularly IFN-γ (fold change = 6.55). **b** Total RNA was prepared from HT29 human colon carcinoma cells and CT26.WT mouse colon carcinoma cells which had been treated with different concentrations of IFN-γ. The qTR-PCR results showed that treatment with IFN-γ increased IRF-1 expression at the mRNA level in both cell lines, whereas IRF-2 expression did not show a noticeable increase. **c** Total proteins were extracted from the pre-treated CRC cells and analyzed in western blot studies that used antibodies against IRF-1 and IRF-2. Histone was used as a loading control. **c** The differential expression of IRF-1 and IRF-2 in the liver specimens was also tested using IHC methods. The levels of IRF-1, but not IRF-2, were dramatically increased in the inflamed liver specimens, and IRF-1-positive structures were located mainly in regions where metastatic tumors were present. **e** Results from qRT-PCR studies showed that let-7a cluster expression was dramatically downregulated in both CRC cell lines after the cells had been treated with different concentrations of IFN-γ for 48 h (*P* < 0.05). A two-tailed *t*-test was used to compare data obtained from the qRT-PCR analyses of two independent groups. Values are shown as the mean ± SD. *P < 0.05; **P < 0.001

Increased levels of pro-inflammatory factors such as IFN-γ, TNF-α, and interleukin (IL-6, IL-1β) were released into the blood while creating our models of chronic liver disease, and played critical roles in the resistance to CRLM^37,38^. In this study, we used enzyme-linked immunosorbent assay (ELISA) methods to detect those factors in liver specimens and corresponding serum samples from mice in the inflammation and control groups (Fig. 2a). In the group with hepatitis and also the normal group, there were some slight differences in the expression of those three factors in liver specimens, but the differences did not reach statistical significance. However, the levels of TNF-α (*P* < 0.05), IL-1β (*P* < 0.01), and particularly IFN-γ (6.55-fold increase, *P* < 0.01) were significantly increased in the serum of mice with hepatitis.

In order to verify the critical role of IFN-γ in mediating this resistance process, the IFN-γ receptor 1 (IFNGR1) found in CT26.WT cells was knocked down by transfection with lentiviral vectors (shRNA-IRFGR1) for 48 h; after which, the shRNA-IRFGR1-CT26 cells and control cells were injected into BALB/c mice (*n* = 10) with hepatitis (Supplementary Figs. 1A–D). Liver metastases were found in eight mice in the IFNGR1 knockdown group and three mice in the control group (80% vs. 30%, *P* = 0.072). Furthermore, more powerful fluorescence (*P* < 0.01), more metastatic foci (*P* < 0.01), and larger maximum metastatic tumor diameters (*P* < 0.01) were also detected in the IFNGR1 knockdown group. These findings suggest that IFN-γ plays an important role in determining the lower incidence of CRLM in a CCl_4_-induced inflammatory environment.

### There was an inverse correlation between expression of the let-7a cluster and IRF-1 in the liver inflammatory environment

Based on information in the UCSC website, numerous interferon regulatory factor (IRF-1 and IRF-2) predicted binding sites (PBS) were found to be located at 5–50 kb upstream of the let-7a cluster transcription start site (TSS) (Supplementary Fig. 2). Because previous studies had showed that IRF-1/2 expression could be regulated by IFN-γ via the IFNGR/JAK/STAT pathway^39,40^, we hypothesized that IFN-γ, mediated by IRF-1 or IRF-2, might inhibit let-7a cluster gene expression in an inflammatory microenvironment.

To test our hypothesis, HT29 human colon carcinoma cells and CT26.WT mouse colon carcinoma cells were cultured and treated with different concentrations (20, 40, 60, 80, and 100 ng/mL) of recombinant human IFN-γ and recombinant murine IFN-γ, respectively, for 48 h; after which, the expression levels of IRF-1 and IRF-2 mRNA were detected by quantitative real-time reverse transcription polymerase chain reaction (qRT-PCR) (Fig. 2b). The results showed that IRF-1 expression in both cells lines was significantly increased following stimulation with increasing concentrations of IFN-γ for 48 h. However, there was no induction of IRF-2 expression in either cell line. Next, HT29 cells and CT26.WT cells were each treated with 40 and 80 ng/mL of IFN-γ, and western blot analyses with anti-IRF-1 and anti-IRF-2 antibodies were performed to detect IRF-1/IRF-2 protein expression in the IFN-γ-treated cell lines (Fig. 2c). A trend toward significantly higher levels of IRF-1 expression, without higher levels of IRF-2 expression, was found in the IFN-γ-treated groups of both cell lines. Furthermore, an immunohistochemical (IHC) analysis that was performed to detect the localization of IRF-1/IRF-2, and semi-quantify its expression in metastatic liver specimens, showed the same results (Fig. 2d). IRF-1 expression was dramatically increased in the CCl_4_-induced inflammation group, and IRF-1-positive structures were located mainly in the areas where metastatic tumors existing.

In parallel with these experiments, we also observed a trend toward lower levels of let-7a cluster expression in the inflammation group. The clustered miRNAs as detected by qRT-PCR performed (Fig. 2e) with specific primers were dramatically downregulated in response to treatment with IFN-γ for 48 h in both CRC cell lines, and especially in the HT29 human colorectal cancer cell line.

### IRF-1 mediated the IFN-γ/let-7a-1-5p pathway

**Fig. 3:**
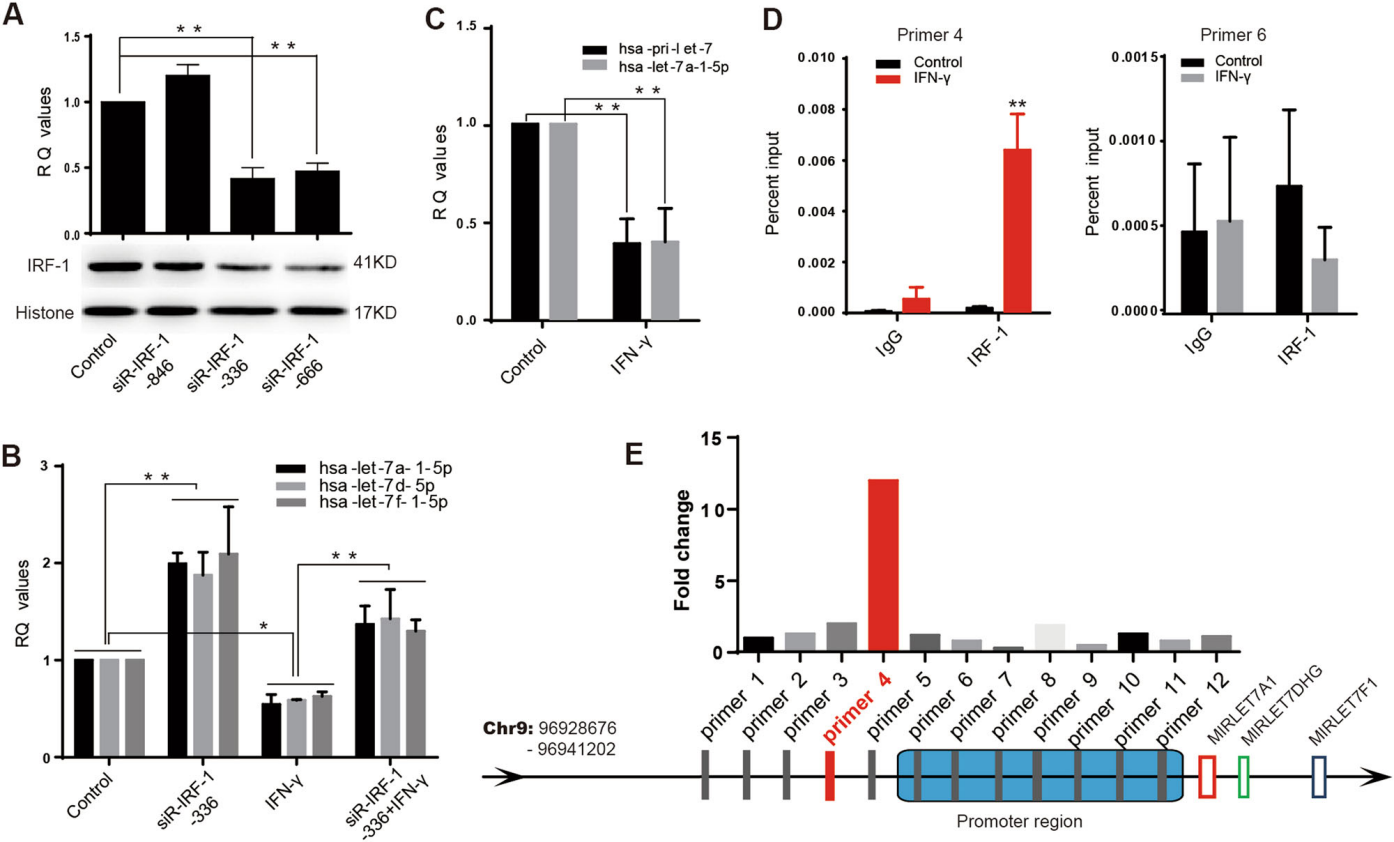
IRF-1 mediated the downregulation of let-7a cluster by stimulating with IFN-γ. **a** Three siRNA-IRF-1 (−846, −336, −666) were transfected in HT29 cell line, qRT-PCR and western blot was used to test the interference efficiency, and siR-IRF-1-366 was filtered out to be the most efficient one (*P* < 0.01). **b** IRF-1 was depleted in HT29, followed by treatment with IFN-γ. The IFN-γ induced decreasing trend of let-7a cluster was significantly attenuated by siRNA-IRF-1-366 transfection (*P* < 0.01). **c** The expression level of let-7a-a-5p and its precursor was significantly decreased under the treatment of IFN-γ (all value *P* < 0.01). The decreased trend of let-7a-1-5p was consistent with its precursor (*P* > 0.05). **d**, **e** ChIP assay was used to find out the possible binding sites of IRF-1 in the upstream of let-7a cluster transcription start site (TSS). The prepared chromatin was immunoprecipated through antibodies against IRF-1 and IgG. And the subsequent qRT-PCR was used to test those possible binging sites with specific primers. The results told that the possible binding site (chr9:96,931,077–96,931,229) was strongly increased amplification with primer #4. A two-tailed *t*-test was used to test the data obtained from the qRT-PCR

Considering the bio-informatic analysis results (Supplementary Fig. 2), we hypothesized that the let-7a cluster downregulation induced by IFN-γ had been mediated by IRF-1 in both CRC cell lines. Rescue experiments were performed to further explore this hypothesis. Small interfering RNAs (siR-IRF-1-864/-336/-666) were used to inhibit IRF-1 expression in HT29 cells; after which, qRT-PCR and western blot studies were performed to detect the respective interference efficiencies (Fig. 3a). We found that the mRNA and protein expression levels were dramatically decreased following transfection with either siR-IRF-1-336 (*P* < 0.01) or siR-IRF-1-666 (*P* < 0.01) for 48 h, with siR-IRF-1-336 producing the stronger inhibitory effect. We next exposed HT29 cells in which IRF-1 had been knocked down to IFN-γ for 48 h, and monitored let-7a cluster expression by qRT-PCR. We found that IFN-γ-mediated repression of the let-7a cluster was significantly reduced in the cells in which IRF-1 had been knocked down (*P* < 0.01) (Fig. 3b). This suggested that IFN-γ-induced downregulation of the let-7a cluster might be mediated by IRF-1.

In order to study the detailed mechanisms by which IRF-1 regulates the let-7a cluster, the expression levels of let-7a-1-5p and its corresponding precursor in HT29 cells were examined by qRT-PCR. A marked downregulation of both mature miRNA (*P* < 0.05) and precursor miRNA expression (*P* < 0.05) was detected in IFN-γ-treated HT29 cells, and the trends of expression were not significantly different (*P* > 0.05) (Fig. 3c). This indicated that depletion of the let-7a cluster was mediated in via transcriptional regulation.

Chromatin immunoprecipitation (ChIP) was performed to detect the specific binding site for IRF-1 upstream of the let-7a cluster TSS. Antibodies against IRF-1 and IgG were used to immunoprecipitate chromatin prepared from normal and IFN-γ-treated HT29 cells (Fig. 3d,e); qRT-PCR was performed with 13 different primers to test the possible binding sites (PBS) (Supplementary Table 2). Results showed that the PBS (chr9:96,931,077–96,931,229) which most strongly increased amplification with primer #4 was located in the upstream region of the TSS, ~7157 bp from the start site.

**Fig. 4:**
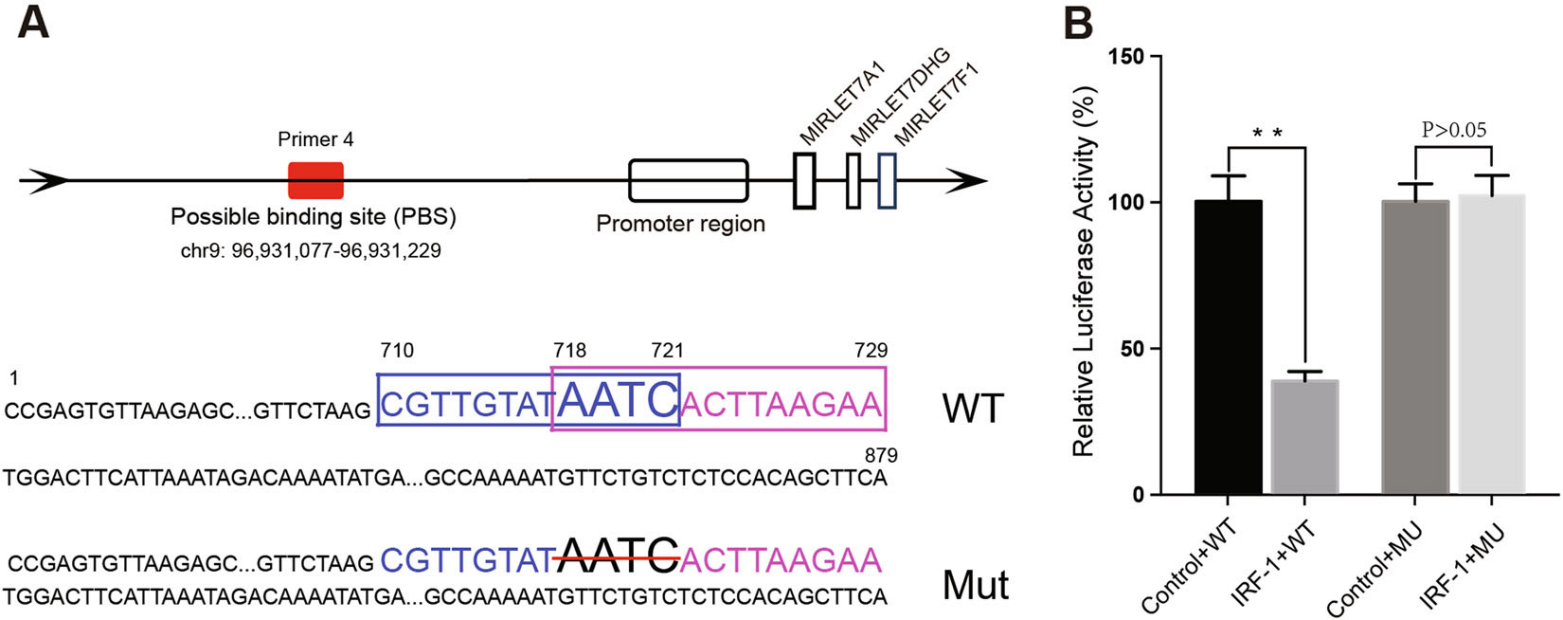
Dual-luciferase reporter assay system. **a** The constructs of upstream of the let-7a cluster TSS (879 bp) and the spot of deletion mutation at position 718–721. The constructs contained the PBS of IRF-1 (chr9: 96,931,077–96,931,229) which was the most strongly enriched amplification with primer #4. The blue box and pink box showed the predicted binding site from the search results of UCSC website. **b** The relative luciferase activities after transfection with different reporter vectors and IRF-1 plasmid. The results showed that the mean level of luciferase activity was significantly lower in the IRF-1 plasmid and let-7a-Report-WT co-transfected group than that in the control plasmid and let-7a-Report-WT co-transfection group (*P* < 0.01). While the let-7a-Report-MU co-transfected with IRF-1 plasmid did not reach the similar statistic difference in luciferase activity (*P* > 0.05). A two-tailed *t*-test was used to test the luciferase activities in different groups. *P < 0.05, **P < 0.01

Additional dual-luciferase reporter assays were performed to support our hypothesis. The constructs upstream of the let-7a cluster TSS (879 bp), the PBS (chr9: 96,931,077–96,931,229), and the spot of the deletion mutation at position 718–721 (AATC) are shown in Fig. 4a. The luciferase activity assay (Fig. 4b) showed that the mean level of luciferase activity in the IRF-1 plasmid and let-7a-Report-WT co-transfected group was significantly lower than that in the control plasmid and let-7a-Report-WT co-transfected group (*P* < 0.01). Co-transfection with the IRF-1 plasmid and let-7a-Report-MU caused no significant change in luciferase activity when compared with the corresponding effects produced by co-transfection with the negative control plasmid and let-7a-1-Report-MU (*P* > 0.05).

### Let-7a-1-5p expression levels were related to colorectal cancer liver metastasis in vivo

**Fig. 5:**
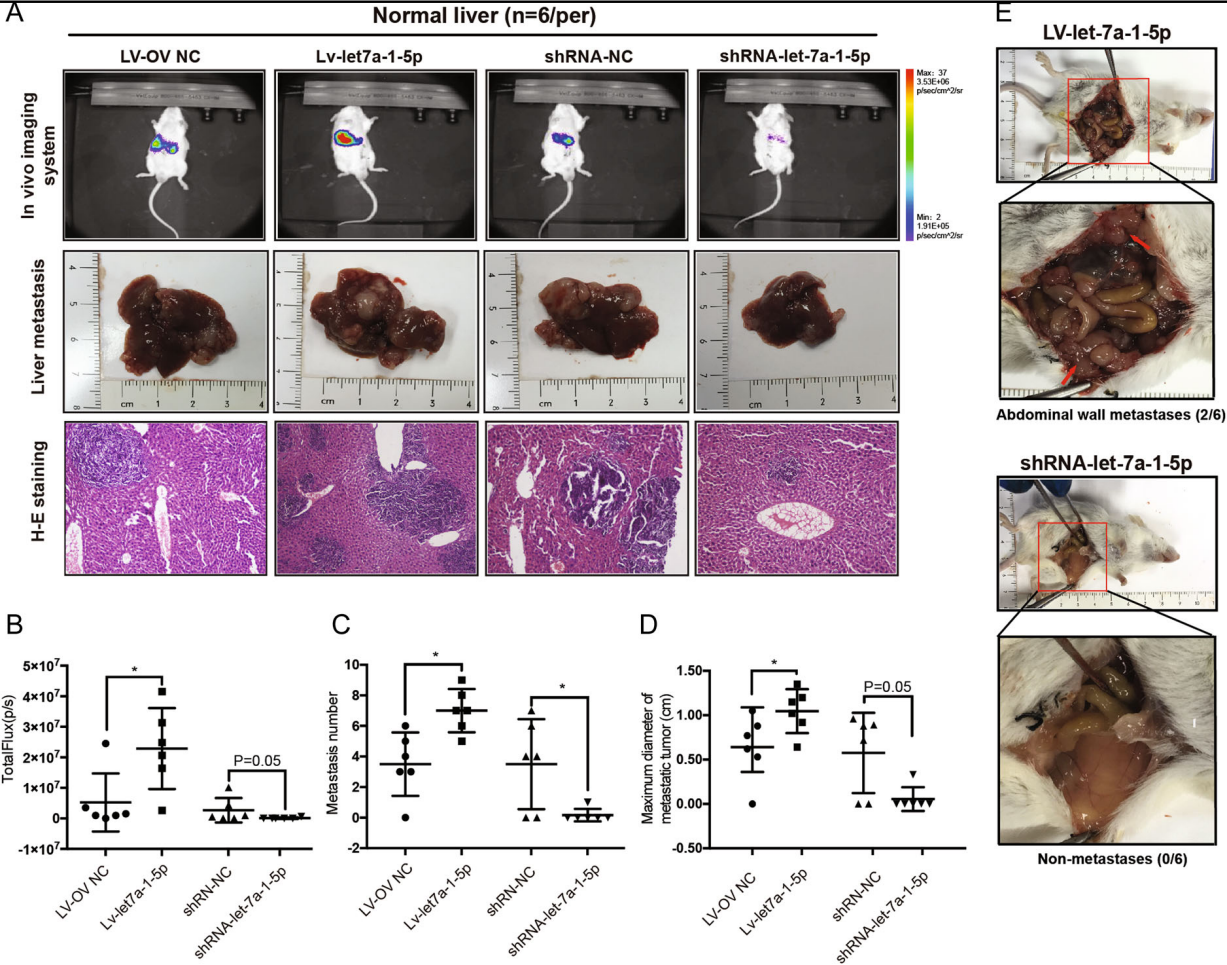
Let-7a-1-5p influenced the metastatic ability of CT26.WT in BALB/c. **a,** **b** Twelve days after the injection of reconstructed CT27 cells, the whole-body in vivo imaging system was used to monitor the dynamic growth of the metastases in BALB/c mice. Compared to the control group (*n* = 6), a lower incidence of CRLM (16.7% vs. 4/6, *P* = 0.242) with less powerful fluorescence (*P* = 0.05) was detected in the let-7a downregulated group (*n* = 6), and more powerful fluorescence in the upregulated group (*P* < 0.05). However, the incidence of CRLM in the overexpression group (*n* = 6) did not achieve statistical significance (6/6 vs. 5/5) compared to the control group (*n* = 6). Macro- and microscopic changes were observed after performing anesthesia and hepatectomies. More discrete tumor nodules were visible throughout the livers in the upregulated group, and it was identical with histological and morphological characteristic observed from H&E stain. **c**, **d** The number and the maximum diameter of metastases in different groups. Dramatically decreased trends in number (*P* < 0.05) and diameter (*P* = 0.05) were detected in the let-7a-1-5p suppressive group compared with the control group and increased number of metastases (*P* < 0.05) and dramatically larger diameter (*P* < 0.05) in the upregulated group. **e** Abdominal wall metastases (where the red arrow is pointing) were detected in the two of six mice in the let-7a-1-5p overexpression group. However, no abdominal wall metastasis was found in the other groups. Chi-square test was used to test the rates of incidence of metastasis. Nonparametric test was used to test the total powerful of fluorescence, the number of metastatic foci, and the maximum diameter of metastasis. *P < 0.05, **P < 0.01

Stable overexpression and knockdown of let-7a-1-5p in CT26.WT cells were established using lentiviral vectors according to the manufacturer’s instructions. Metastatic models were constructed by injecting CT26 cells into the spleens of BCLB/c mice for 12 days. Whole-body in vivo imaging and hepatectomy were performed to investigate the incidence of CRLM (Fig. 5a–d). The results showed that let-7a-1-5p plays a critical role in determining the incidence of CRLM in mice. When compared with mice in the control group, mice in the let-7a-1-5p depletion group had a lower incidence of CRLM (16.7% vs. 66.7%, *P* = 0.242), less powerful fluorescence (*P* = 0.05) during imaging (Fig. 5b), fewer metastatic foci (*P* < 0.05) in their liver specimens (Fig. 5c), and smaller maximum diameters (*P* = 0.05) of their metastatic tumors (Fig. 5d). Although a slightly higher incidence of CRLM was found in the upregulated group (100% vs. 83.3%), the increase was not statistically significant (*P* > 0.05). Mice in the upregulated group also displayed more powerful fluorescence (*P* < 0.05), larger numbers of metastatic foci (*P* < 0.05), and larger maximum tumor diameters (*P* < 0.05) when compared with control mice. In addition, abdominal wall metastases were only detected in the overexpression group (2/6 mice vs. 0/6 mice) (Fig. 5e).

Splenic injection of these constructed CT26 cells transfected with shRNA-let-7a-1-5p or LV-let-7a-1-5p was also performed in mice with hepatitis (*n* = 10) (Supplementary Figs. 1E–H). The incidence of CRLM in the former group was 10%, compared to 50% in the latter group (*P* = 0.141). Furthermore, differences in the incidence of powerful fluorescence, the number of metastatic foci, and the maximum diameter of metastatic tumors all reached statistical significance (all *P*-values <0.05).

### Repression of the let-7a cluster maintained the mesenchymal phenotype of CRC cells and prevented their subsequent settlement in the liver

Surprisingly, some of the HT29 cells, but no CT26.WT cells, reversed from their epithelial phenotype to a mesenchymal phenotype during the process of modeling the let-7a-1-5p depleted cell line with lentivirus. Several studies have shown that abnormal miRNA expression is closely associated with the EMT and migratory capability of cancer cells^33,41–43^. Furthermore, Peter et al. found that downregulation of let-7 promotes the EMT^44–46^.

**Fig. 6:**
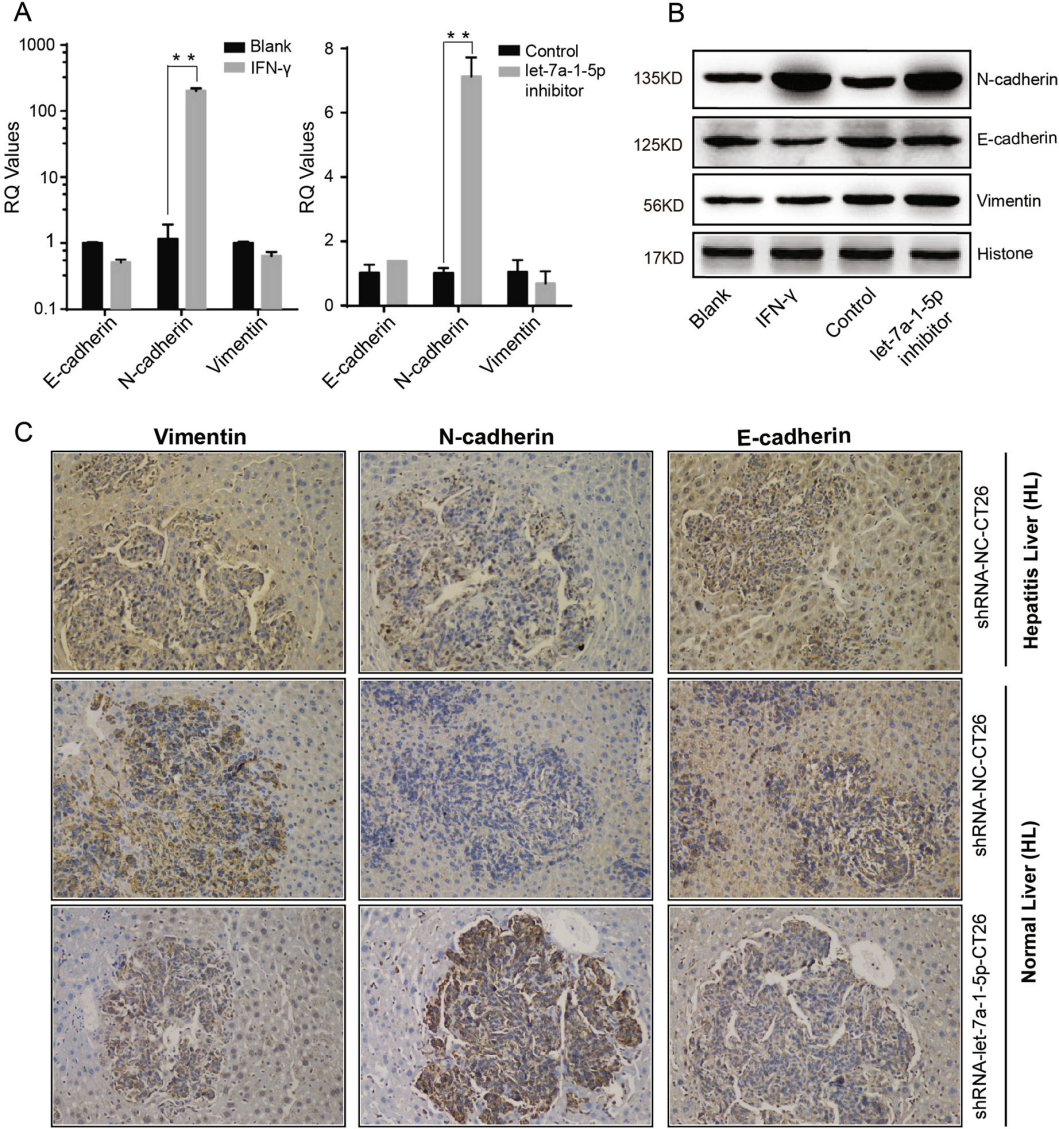
Repression of let-7a cluster maintained the mesenchymal phenotype of CRC cells. **a**, **b** IFN-γ stimulation and let-7a-1-5p depletion induced a significant up-regulation of N-cadherin in HT29 cells in mRNA and protein level, but not E-cadherin and Vimentin. **c** The expression of those three EMT relative markers in liver specimens sampled from the metastatic animal models were detected by immunohistochemical staining (IHC). N-cadherin were dramatically upregulated in the inflammatory group and the let-7a-1-5p depleted group, especially in the tumor areas. However, the differential expression of E-cadherin and Vimentin in the inflammatory and let-7a-1-5p depleted groups was not statistically significant. A two-tailed *t*-test was used to test the data obtained from the qRT-PCR analyses. *P < 0.05; **P < 0.001

In this study, EMT related markers such as E-cadherin, N-cadherin, and vimentin were investigated both in vitro and in vivo. Our data from qRT-PCR studies showed that in HT29 cells, expression of N-cadherin, a known marker of the mesenchymal phenotype, was dramatically increased by >100-fold after stimulation with IFN-γ (*P* < 0.01), and by >7-fold after depletion of let-7a-1-5p in HT29 (*P* < 0.01); however, the other two markers showed no significant differential expression trend (Fig. 6a). Moreover, our western blot studies showed the same results (Fig. 6b).

Our IHC studies of liver specimens revealed that N-cadherin levels were markedly increased in both the inflammation group and the let-7a-1-5p downregulated group, when compared with their respective control groups (Fig. 6c). N-cadherin-positive structures were located mainly areas infiltrated with tumor cells.

**Fig. 7:**
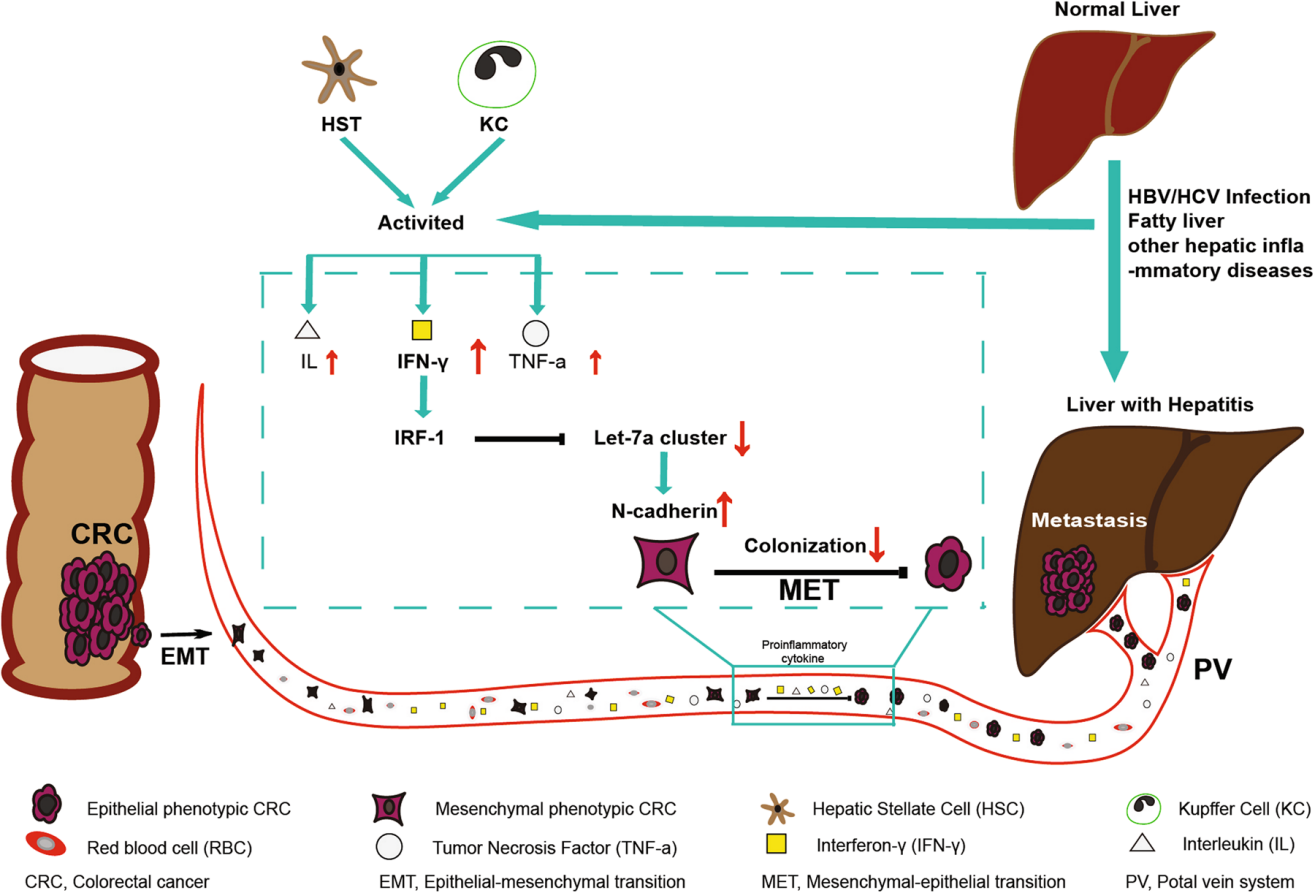
A schematic model of the critical role of IFN-γ/IRF-1/let-7a-1-5p cluster/EMT pathway in regulating the subsequent colonization of circulating colorectal cancer cells to livers with inflammatory microenvironment. Activation of hepatic stellate cells and Kupffer cells often followed the HBV/HCV infection or other hepatic inflammatory diseases and was characterized by the overwhelming release of pro-inflammatory cytokines such as IFN-γ, TNF-α, and IL into in peripheral circulating blood. IFN-γ was the sharpest upward one. Primary colorectal cancer cells firstly required EMT to gain mesenchymal phenotype for the invasive ability and prompt intravasion. The IRF-1 product of those circulating colorectal cancer cells was upregulated by the stimulation of IFN-γ and then dramatically depleted the expression of the let-7a cluster. The suppression of the let-7a cluster caused significantly overexpression of N-cadherin which was associated with acquiring the mesenchymal phenotype. As we all know, MET, a reverse process of EMT, is necessary for facilitating the extravasation and subsequent settlement of circulating colorectal cancer cells at secondary locations. So it is not difficult to understand that maintaining the mesenchymal phenotype of circulating CRC cells might result in low incidence of CRLM

In this study, we confirmed that downregulation of let-7a-1-5p helps to maintain the mesenchymal phenotype of circulating CRC cells by causing them to overexpress N-cadherin, and prevents CRC cells from settling in the liver (Fig. 7).

## Discussion

It has become an indisputable fact that colorectal cancer liver metastases are rarely found in patients with pathological liver conditions such as a hepatitis B or C virus infection, fatty liver, cirrhosis, or other chronic hepatic diseases. However, most studies of this phenomenon have been retrospective clinical studies, and did not yield insights into the pertinent molecular mechanisms.

In this study, we first demonstrated the inhibitory effect of liver inflammation on CRLM in vivo. Animal models of inflammation and CRLM were used to confirm that circulating CRC cells, transplanted by splenic injection, rarely developed distant metastases in inflamed livers (83.3% vs. 33.3%, *P* = 0.079), which resulted in significantly lower tumor burdens in those livers. Hepatitis results from sustained injury produced by chronic hepatic diseases, and is characterized by collagen deposition and the accumulation of extracellular matrix macromolecules. Kupffer cells (KCs), natural killer (NK) cells, and cytotoxic T lymphocytes (CTLs) are all activated during this process.

NK cells and CTLs play crucial roles in the immune response to a tumor, and prevent cancer cell growth and distance dissemination^47,48^. The infiltration of NK cells into colorectal cancer tissue has been associated with a favorable clinical outcome^49,50^. The loss of MHC class I expression makes metastasizing CRC cells particularly vulnerable to destruction by circulating for NK cells, and thus inhibits the development of distant metastases^51–53^. NK cells can also prevent metastasizing CRC cells from developing a hepatic metastasis focus by acting through the Fas/Fasl pathway and Perforin/Granzyme pathway. CTLs are a specific type of T cell that can damage metastatic CRC cells, and also secrete various cytokines that enhance their antitumor function.

Activated KCs exposed to an inflammatory environment can release increasing amounts of profibrogenic factors such as IFN-γ, TNF-α, and IL. These factors can induce the activation of hepatic stellate cells, and play important roles in the development of chronic liver diseases. In this study, ELISA was used to examine the expression levels of those cytokines in serum specimens and liver tissues of mice. The results indicated that a significantly increased serum IFN-γ level may serve as an important biomarker for CCl_4_-induced hepatitis (Fig. 2a), and play a primary role in the process by which CRC cells are prevented from metastasizing to inflamed livers^54,55^. To determine whether IFN-γ plays a major role in controlling resistance to tumor metastasis in mice with hepatitis, one of the IFN-γ receptors (IFNGR1) was knocked down by shRNA-IRFGR1 transfection. The results showed that IFN-γ plays an important role in determining the lower incidence of CRLM in inflammatory environment.

When combined with our previous studies, the ChIP assay and dual-luciferase reporter assay results mentioned above indicate that IRF-1 mediates downregulation of the let-7a cluster by binding to the upstream region of the let-7a cluster TSS.

Several studies have shown that the let-7a cluster, a cancer suppressor gene, inhibits tumor cell proliferation, invasion, distant colonization, and the EMT. Depletion of let-7a cluster miRNA might result in tumor progression. Moreover, studies have also shown that the let-7a cluster is dramatically upregulated in colorectal cancer tissue^56^ and is closely related to a poor clinical prognosis and the presence of hepatic metastases^57^. Therefore, we hypothesized that a lower incidence of CRLM might result from decreased expression of let-7a cluster in normal and inflammatory liver microenvironments.

CT26 cell lines with either stable overexpression or knockdown of let-7a-1-5p were used to test this hypothesis in normal BALB/c mice. Depletion of let-7a-1-5p led to a lower incidence of CRLM and a dramatically lower tumor burden. In contrast, mice injected with LV-let-7a-1-5p-CT26 cells had a slightly higher incidence of CRLM, and displayed significantly more powerful fluorescence. Those mice also had more metastatic foci, tumors with larger maximum diameters, and were more likely to have abdominal wall metastases (Fig. 4d). Our subsequent studies in inflammatory BALC/c mice showed similar results (Supplementary Fig. 1).

Depletion of the let-7a cluster maintained the mesenchymal characteristics of circulating CRCs and inhibited their hepatic settlement in the inflammatory microenvironment. It is generally known that HT29 cells, an epithelial phenotypic CRC cell line, are characterized by their ability to adhere to each other and their 3D structural growth patterns, and that those characteristics prevent HT29 vicariance. However, this epithelial phenotype became transformed into a mesenchymal phenotype when let-7a-1-5p was depleted. In subsequent experiments, extremely high levels of N-cadherin, a molecule associated with acquisition of the mesenchymal phenotype, were detected in IFN-γ-treated and let-7a-1-5p-depleted cells by qRT-PCR, western blot, and IHC.

How could the EMT, known as a promoter of metastasis, be triggered by decreased expression of let-7a cluster, and even prevent CRLM in an inflammatory microenvironment? It is known that both the EMT process and mesenchymal–epithelial transition (MET) process (a reverse of EMT) play key roles in CRLM formation, which requires a series of distinct steps involving local invasion, intravasation, dissemination in the circulation, extravasation, colonization, and metastasis formation^58^. Polyak et al. clarified in detail the phenotypic changes that occur in primary cancer cells during the entire process of distant metastasis. Primary cancer cells must first complete the EMT process to gain a mesenchymal phenotype required for their invasive ability and prompt intravasation; after which, the MET is necessary for facilitating their extravasation and subsequent settlement at secondary locations^59^. Other than for those requirements, the clinicopathological properties of primary colorectal cancer are quite similar in HBV-infected and non-infected patients, and the low incidence of CRLM in inflamed livers is not caused by the modulation of pathological factors in primary CRC tumors^10,13^. In this study, transfected CT26 cells were injected into the splenic vein and became circulating tumor cells, which ultimately reached the liver via the portal vein. Therefore, it is not a contradiction to infer that the loss of let-7a cluster expression prevented disseminated circulating CT26 cells from subsequently settling in the livers of mice with CCl_4_-induced hepatitis. Understanding details of the mechanism by which depletion of let-7a cluster maintains the mesenchymal phenotype of CRC cells is worthy of further study.

In summary, we identified the critical role played by the IFN-γ/IRF-1/let-7a cluster/N-cadherin pathway in regulating the subsequent settlement of circulating CRC cells into livers with an inflammatory microenvironment.

However, our study has some deficiencies that should be mentioned. Hepatitis has variety of etiologies that include infection with HBV/HCV, fatty liver, autoimmune hepatitis, and other chronic liver diseases. The CCl_4_-induced animal model of liver inflammation is not entirely representative of the liver inflammation found in hepatitis patients. In addition, although the incidence of metastasis obviously differed in the different groups, further studies with larger sample sizes are needed for these differences to reach statistical significance. Therefore, it is imperative that we establish new types of animal models and perform the in-depth research needed to verify the conclusion mentioned above.

## Materials and methods

### Animal models

Male BABL/c mice (6 weeks old) were purchased from the Laboratory Animal Center of the Second Military Medical University (Shanghai, China). All the animal experiments were conducted according to the National Institutes of Health animal use guidelines with methods of random grouping. The hepatic inflammatory animal models were constructed by gavaging 40% CCl4 (6 mL/kg), age-matched male BALB/c who received saline gavage as controls. To evaluate incidence of metastasis in vivo, we firstly weighed the mice and injected 100 μL chloralhydrate (10%) into the abdominal cavity for anesthesia. 0.2 mL reconstructed CT26 cell suspension (1 × 10^6^/per mouse) was injected into the spleen and splenectomy was performed to stop the cancer cell from growing in the spleen. The incidence of CRLM was detected using the whole-body in vivo imaging system with intraperitoneal injection of luciferin (Promega, USA) and subsequent hepatectomy was performed followed the principles of aseptic surgery.

### Cell culture

Two CRC cell lines (HT29 and CT26.WT) were purchased from the American Type Culture Collection (ATCC, Rockville, MD, USA). All the cell lines were stored and cultured according to the instructions of ATCC. The HT29 was cultured in McCoy’s 5a (SIGMA, M4892, USA) and CT26.WT in RPMI-1640 (Hyclone, China) containing 10% fetal bovine serum (Gibco, 10099-141, Australia) at 37 °C in a humidified incubator supplemented with 5% CO_2_.

### RNA extraction and real-time PCR

Total RNA was extracted from the tissues and cell using RNAiso Plus kit (TaKaRa, AA1003-1, Japan), then 1.0 μg total RNA was reverse transcribed into cDNA using ReverTra Ace qPCR RT Kit (TOYOBO, FSQ-101, Japan) with a universal primer (5′-AACGCTTCACGAATTTGCGT-3′) for mRNA and a specific miRNA stem loop primer (5′-GTCGTATCCAGTGCGAACTGTGGCGATCGGTACGGGCTACACTCGGCAATTGCACTGGATACGACAACTA-3′) for let-7a cluster. The mRNA and miRNA expression levels were quantitatively measured by SYBR Green-based qRT-PCRs with gene-specific primers; GAPDH and U6 were used as the endogenous control, respectively. All qRT-PCRs were conducted using SYBR® Green Real-time PCR Master Mix (Toyobo, QPK-201, Japan) and repeated three times. The primers are listed in Supplementary Table 1.

### Western blot analysis

HT29 and CT26.WT were cultured in a six-well plate for 48 h; total protein was extracted using RIPA buffer (Servicebio, G2002, China) containing protease inhibitors (Servicebio, G2006, China) and quantified using BCA kit (Thermo Fisher Scientific, USA). For each sample, 25 μg total protein was separated by 10% SDS-PAGE and transferred to a PVDF membrane using a wet transfer blotting system (BioRad, Hercules, CA), antibodies such as anti-IRF-1 (Cell Signaling Technology, #8474, USA), anti-IRF-2 (Abcam, ab1274744, USA), anti-E-cadherin (ABclonal, A3044, China), anti-N-cadherin (ABclonal, A0433, China), anti-Vim (ABclonal, A11423, China) were used for western blotting; anti-histone (Abcam, USA) was used as an endogenous control.

### Transfection procedure

Optimal concentration of let-7a cluster inhibitors (GenePharma, China), small interfering RNA (siRNA) targeting IRF-1(siR-IRF-1) (Genechem, China) and their corresponding negative control (NC) were transfected into CRCs with Lipofectamine^TM^ 2000 Transfection Reagent (Invitrogen, USA). Forty-eight hours after transfection, total RNA, and protein were collected and subsequent assays was performed on schedule. Lentiviral vector (pLenti-U6-hsa-let7a-1-EF1a-LUC-T2A-Puro) (Obio, China) was used to overexpress let-7a-1-5p (LV-let-7a-1-5p) and pLenti-U6-mir30-EF1a-LUC-T2A-Puro H130 to silence let-7a-1-5p (shRNA-let-7a-1-5p) expression in CT26 and HT29 cell lines. After 3 days' transfection, LV-let-7a-1-5p-CT26 and shRNA-let-7a-1-5p-CT26 were cultured in medium containing puromycin (0.5 μg/mL) for 2 weeks, and stable overexpression and depletion of let-7a-1-5p in CT26 were selected. All transfection procedures were performed according to the manufacturer’s instructions.

### ChIP assay

ChIP assays were performed using the EZ ChIP^TM^ Chromatin Immunoprecipitation Kit (Millipore, 17–371). HT29 (1 × 10^7^) cells were treated with IFN-γ (50 ng/mL) for 48 h. Two hundred and twenty-five microliters 37% formaldehyde were added to crosslink protein and DNA at room temperature. Then the crosslinked cells were scraped from the dish, lysed by SDS lysis buffer, and sonicated to 200–500 bp DNA on optimal condition. The crosslinked protein/DNA complexes were immuneprecipitated with specific anti-IRF-1antibody (Abcam, ab186384, USA) and reversed by incubating with 5 M NaCl at 65 °C for 5 h. At last, the free DNA was purified and the special-gene was quantified using qRT-PCR with 13 specific primers which complement to the promoter region of let-7a cluster. All the primers are listed in the Supplementary Table 2.

### Dual-luciferase reporter assay system

The constructs of upstream of the let-7a cluster TSS (879 bp) were amplified by PCR with special primer (the sense primer 5′-CCGAGTGTTAAGAGCGCCA-3′ and the anti-sense primer 5′-TGAAGCTGTGGAGAGACAGAAC-3′) using the genomic DNA from HT29 cells. This region contained the possible IRF-1 binding site (chr9: 96,931,077–96,931,229). The UCSC predicted binding sites, which located at positions 710–721 (CGTTGTAT**AATC**) and 718–729 (**AATC**ACTTAAGA), and the deletion mutation was located at position 718–721 (**AATC**). All the constructs were sequenced to exclude additional mutations and then ligated and cloned into multicloning sites of the firefly luciferase pGL4.26-Basic vector according to the instruction of the kit. The mutant reporter plasmid let-7a-Report-MU (at positions 718–721) and IRF-1-expressing plasmid CMV-IRF-1-EGFP-SV40-Neomycin (Genechem, China) were also constructed. The combination of IRF-1 plasmid or its control plasmid with let-7a-Report-WT and the combination of IRF-1 plasmid or its control with let-7a-Report-MU, were co-transfected into HT29 cells. After 48 h, HT29 cells were collected, lysed, and the relative luciferase activity was measured.

### H&E stain and IHC staining

All the tissue was fixed in 10% formaldehyde, dehydrated in ethanol, cleared in xylene, and embedded in paraffin. Sections (5 μm) were subjected to routine H&E stain and established the IHC staining technique using standard procedures. Rabbit mAb to IRF-1 (Cell Signaling Technology, #8474, USA), IRF-2 (Abcam, ab124744, USA), E-cadherin (ABclonal, A3044, China), N-cadherin (ABclonal, A0433, China), and Vimentin (ABclonal, A11423, China) were used as the primary antibodies and HRP Goat anti-rabbit IgG (ABclonal, China) was used as the secondary antibody.

### Statistical analysis

Data were presented as the mean ± SD at least three independent experiments. A two-tailed *t*-test was used for comparisons of the data which were obtained from the qRT-PCR analyses of two independent groups. Chi-square test was used to test the rates of incidence of metastasis. Nonparametric test was used to test the total powerful of fluorescence, the number of metastatic foci, and the maximum diameter of metastasis. *P* < 0.05 was considered statistically significance (*P < 0.05, **P < 0.01).

## Electronic supplementary material

Supplementary table 1

Supplementary table 2

Supplementary figure 1

Supplementary figure 2

Supplementary Figure Legends
