# Supplementary material for: Repression of let-7a cluster prevents adhesion of colorectal cancer cells by enforcing a mesenchymal phenotype in presence of liver inflammation
Source: Cell Death Dis. 2018 Apr 25;9(5):489. doi: 10.1038/s41419-018-0477-1 (PMC5916926; doi:10.1038/s41419-018-0477-1)
Supplement: Supplementary file 1 — Supplementary table 1 [file 41419_2018_477_MOESM1_ESM.doc]

Supplementary table 1. Primers for qRT-PCR

| Primers | Forward | Reverse |
| --- | --- | --- |
| Hsa- IFN-γ | TCGGTAACTGACTTGAATGTCCA | TCGCTTCCCTGTTTTAGCTGC |
| Mmu- IFN-γ | TGCTGATGGCCTGATTGTCTT | GCCACGGCACAGTCATTGA |
| Hsa IRF-1 | ATTTAACAGGCAAGTCCAACTCA | GGCCACCCTTCTTATACTTCACT |
| Mmu-IRF-1 | GCAAACTTCCGTTGTGCCAT | TCGGCTGGACTTGGACTTTC |
| Hsa-IRF-2 | CATGCGGCTAGACATGGGTG | GCTTTCCTGTATGGATTGCCC |
| Mmu-IRF-2 | GATGCTGCCCTTATCCGAAC | TGCTTGATGTGCTTAACTCTCTC |
| Hsa-let-7a-1-5p | CGGCTGAGGTAGTAGGTTGTAT | AGTGCGAACTGTGGCGAT |
| Hsa-let-7d-5p | CCGAGAGGTAGTAGGTTGCAT | AGTGCGAACTGTGGCGAT |
| Hsa-let-7f-1-5p | GCCCGTGAGGTAGTAGATTGTA | AGTGCGAACTGTGGCGAT |
| Hsa-pri-let-7a-1 | CCTGGATGTTCTCTTCACTG | GCCTGGATGCAGACTTTTCT |
| Hsa-N-cadherin | AGCCAACCTTAACTGAGGAGT | GGCAAGTTGATTGGAGGGATG |
| Hsa-E-cadherin | TTGAGAATGAGGTCGGTGCC | TCAGAATGCCCTCGTTGGTC |
| Hsa-Vimentin | GACGCCATCAACACCGAGTT | CTTTGTCGTTGGTTAGCTGGT |
| GAPDH | ATGTTCGTCATGGGTGTGAA | GGTGCTAAGCAGTTGGTGGT |
| U6 | GCTTCGGCAGCACATATACTAAAAT | CGCTTCACGAATTTGCGTGTCAT |
