# Supplementary material for: Repression of let-7a cluster prevents adhesion of colorectal cancer cells by enforcing a mesenchymal phenotype in presence of liver inflammation
Source: Cell Death Dis. 2018 Apr 25;9(5):489. doi: 10.1038/s41419-018-0477-1 (PMC5916926; doi:10.1038/s41419-018-0477-1)
Supplement: Supplementary file 2 — Supplementary table 2 [file 41419_2018_477_MOESM2_ESM.doc]

Supplementary table 2. Primers for ChIP assay

| Primers | Forward | Reverse |
| --- | --- | --- |
| ChIP1 | TCAATCAGACGGCTTCACCA | GCGAAATAAAATGGCGACG |
| ChIP2 | TTGCTGCCAAAACCGAACAAA | TAAGCCCACTTCCTTGTGTGG |
| ChIP3 | TTCACATTCCTTACCCCGCC | TACAACCAGTCACGCCACAG |
| ChIP4 | GCTTTTTGCCTGTGTAGGTGT | AGCCCCAAGTGTATGTGTTG |
| ChIP5 | CATTCCTTACCCCGCCCTTC | CTACAACCAGTCACGCCACA |
| ChIP6 | TTGGACTCTGCCTTCAATCCAC | TTATCCCATGCGAGCAAACAC |
| ChIP7 | TGCCCCCGCTTCAGATGC | TAACCCTAACTCCATAGATACAGAAGC |
| ChIP8 | GAGTTAGGGTTACACCAGCCTCC | CCAGGTAAAGCAGAGAGGACAGAG |
| ChIP9 | CTAATCACACCTTGGTCTTTCTGGT | CCTTAGATTGCTGGTGGGAATG |
| ChIP10 | ATTTCACATTCCCACCAGCAA | CATTCTTTGTAAACTGGTGTGC |
| ChIP11 | GGCTATACAGCCGTCAGCA | AACATACGGCTCAATATAATAGAAT |
| ChIP12 | GCTACCTCCTAAATATGAAGTCTGT | ATCCCATGCGAGCAAACACT |
| ChIP13 | ACAGGACTTTTGGGTTTTGAAGT | TGGTTATCTGAATCCGCTCCT |
