# Supplementary figures and images for: Repression of let-7a cluster prevents adhesion of colorectal cancer cells by enforcing a mesenchymal phenotype in presence of liver inflammation
Source: Cell Death Dis. 2018 Apr 25;9(5):489. doi: 10.1038/s41419-018-0477-1 (PMC5916926; doi:10.1038/s41419-018-0477-1)

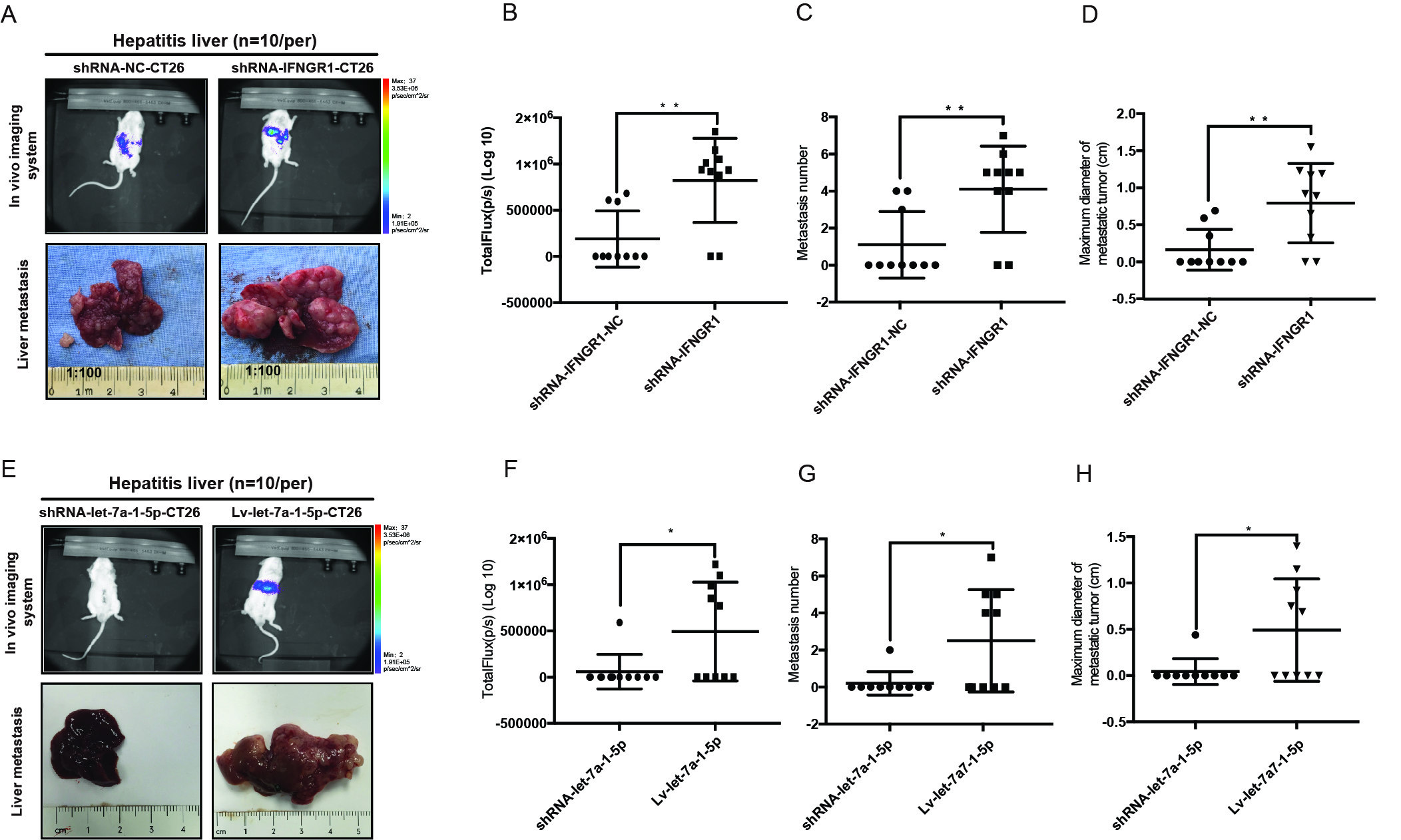

Supplement: Supplementary file 3 — Supplementary figure 1 [file 41419_2018_477_MOESM3_ESM.jpg]

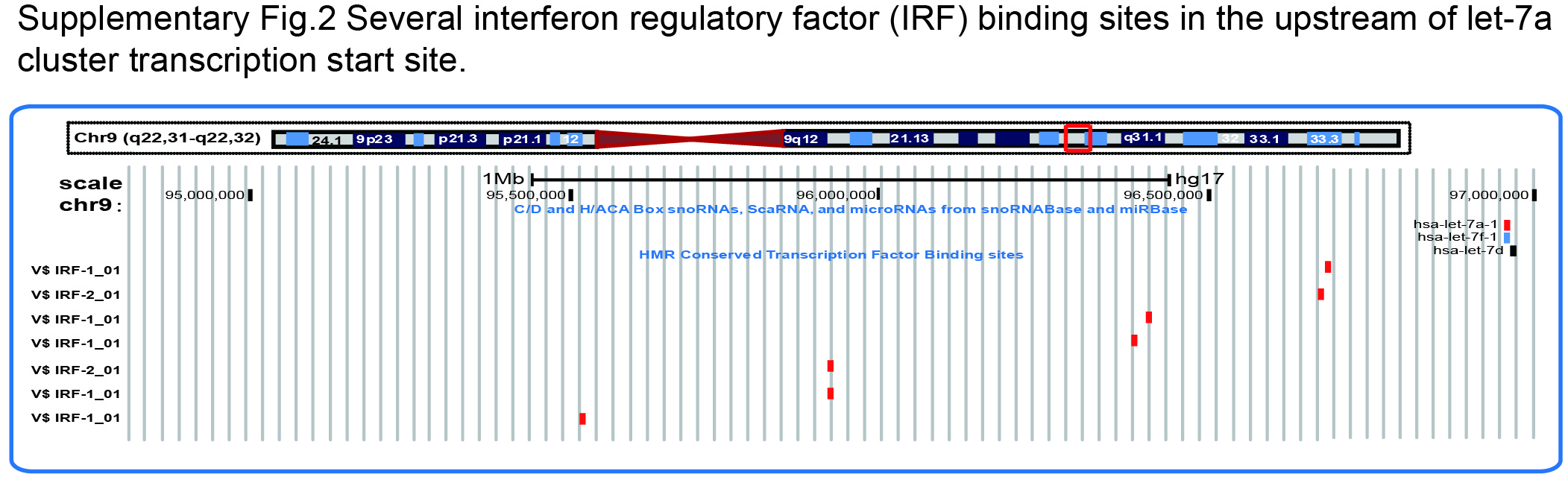

Supplement: Supplementary file 4 — Supplementary figure 2 [file 41419_2018_477_MOESM4_ESM.jpg]
