# Supplementary material for: Repression of let-7a cluster prevents adhesion of colorectal cancer cells by enforcing a mesenchymal phenotype in presence of liver inflammation
Source: Cell Death Dis. 2018 Apr 25;9(5):489. doi: 10.1038/s41419-018-0477-1 (PMC5916926; doi:10.1038/s41419-018-0477-1)
Supplement: Supplementary file 5 — Supplementary Figure Legends [file 41419_2018_477_MOESM5_ESM.docx]

**Supplementary Figure Legends**

**Supplementary Fig.1 (A - D)** **IFN-γ may play critical roles in determining the lower incidence of CRLM in CCl4** **induced inflammatory circumstance.** The IFN-γ receptor 1 (IFNGR1) found in CT26.WT cells was knocked down by transfection with lentiviral vectors, and those shRNA-IRFGR-1-CT26 cells were injected into BALBC/c mice (n = 10) with hepatitis. A higher incidence of CRLM was detected in the IFNGR knockdown group compared with the control group (80% vs. 30%, P = 0.072), and dramatically more powerful fluorescence (P<0.01), more metastatic foci (P<0.01) and larger maximum diameters of metastatic tumor (P < 0.01) were also detected in the IFNGR knockdown group. (E-H) Expression level of the let-7a-1-5p was related to the metastatic ability of CRC cells in liver inflammatory environment. Splenic injection of shRNA-let-7a-1-5p-CT26 cells and LV-let-7a-1-5p-CT26 cells were also performed in the mice with hepatitis (n = 10). The incidence of CRLM in the former group was 10%, compared to 50% in the latter group (P = 0.141), and the total powerful of fluorescence, the number of metastatic foci and the maximum diameter of metastasis also reached the statistical significance (all P-value < 0.05). The Chi-square test was used to test the rates of incidence of metastasis. Nonparametric test was used to test the total powerful of fluorescence, the number of metastatic foci and the maximum diameter of metastasis. * P < 0.05; ** P < 0.001.

**Supplementary Fig. 2 Several interferon regulatory factor (IRF) binding sites in the upstream of the let-7a cluster transcription start site (TSS).** According to the search results from the UCSC ENCODE Genome Browser (http://genome.ucsc.edu), numerous interferon regulatory factor (IRF-1 and IRF-2) predicted binding sites were found to be located at 5-50 kb upstream of the let-7a cluster TSS.
